# Draft Genome Sequence of Hafnia paralvei Strain VBC_1714, Isolated from Frozen Cod Fillet Imported from Russia to Norway

**DOI:** 10.1128/MRA.00624-21

**Published:** 2021-08-19

**Authors:** Julia E. Storesund, Didrik H. Grevskott, Nachiket P. Marathe, Bjørn Tore Lunestad, Cecilie Smith Svanevik

**Affiliations:** a Norwegian Institute of Marine Research, Bergen, Norway; Montana State University

## Abstract

*Hafnia* spp. have the potential to cause opportunistic infections in humans and animals. This announcement describes the draft genome sequence of an H_2_S-positive Hafnia paralvei strain that was isolated as a presumptive Salmonella sp. from a frozen cod fillet.

## ANNOUNCEMENT

*Hafnia* spp. are common gut bacteria that are generally considered nonpathogenic and are associated with cheese production and food spoilage (1). They are, however, infrequently reported as opportunistic pathogens causing infections in humans and animals and have been associated with a range of food products (2, 3). *Hafnia* strains can carry multiple antimicrobial resistance and virulence genes (1, 4–6) and are therefore highly interesting from a human health perspective.

Strain VBC_1714 was isolated from a sample of frozen cod (Gadus morhua) imported from Russia to Norway in 2020 that was analyzed for Salmonella according to ISO:6579-1:2017 (7) as part of the national surveillance of imported seafood. Briefly, 25 g of muscle fillet was aseptically transferred to 225 ml buffered peptone water, homogenized (Stomacher 400 circulator; Seward, UK), and incubated aerobically at 37°C for 18 h. From the incubated enrichment broth, 1 ml was transferred into 10 ml Rappaport-Vassiliadis soy peptone broth (RVS broth) and incubated aerobically at 42°C for 24 h, after which 10 μl was plated on xylose lysine deoxycholate (XLD) agar. One isolate grew as dark pink colonies with a black center and was thus identified as a putative Salmonella strain. *Hafnia* spp. can be mistaken for Salmonella on certain specific growth media (8), but they do not commonly produce H_2_S (9). The strain was purified by restreaking on blood agar and further identified as Hafnia alvei using a Bruker MALDI Biotyper. Genomic DNA was extracted using the Qiagen DNeasy blood and tissue kit (Germany) following the manufacturer’s protocol. The extracted DNA was quantified using a Qubit double-stranded (dsDNA) broad-range (BR) assay kit (Thermo Scientific), and sequencing libraries were prepared using a 2S Turbo DNA library preparation kit (Swift Biosciences, USA). Sequencing was performed using the MiSeq platform (Illumina, USA) with 2 × 300-bp sequencing technology at the Norwegian Sequencing Centre (Oslo University Hospital, Norway).

The barcodes were removed from 1,488,640 raw reads and the reads were quality trimmed using BBDuk v38.63 (10). The contigs were assembled from 1,260,024 quality-filtered reads using SPAdes v3.13.1 with default parameters (11). The assembled genome had an *N*_50_ value of 581,084, a total length of 4,558,914 bp, and a G+C content of 48.1% and consisted of 29 contigs (>500 bp; average coverage, 17.1×), 4,428 coding DNA sequences, 15 rRNAs, and 78 tRNAs. Strain VBC_1714 was further identified as Hafnia paralvei using Kraken2 v2.0.8-beta (12). Genome annotation was performed using the NCBI Prokaryotic Genome Annotation Pipeline v5.0 (13).

The genome of strain VBC_1714 contained two genes associated with H_2_S production in E. coli, the 3-mercaptopyruvate sulfurtransferase gene, which has been associated with H_2_S production under aerobic conditions (14, 15), and *IscS* cysteine desulfurase, which is associated with H_2_S production under anaerobic conditions (14), as well as with sulfur and iron assimilation and Fe-S assembly (16).

Strain VBC_1714 contained one CRISPR array with five spacers and the *arnA* gene, which has been linked to polymyxin resistance (1). The class C β-lactamase gene *bla*_ACC-1b_, which confers resistance to aminopenicillins and narrow-spectrum cephalosporins (4), was identified using AMRFinder v3.1.1b (17). Four complete and two partial prophages were identified using PHASTER (18). One plasmid of 5,216 bp was identified and displayed 100% nucleotide identity to plasmid pAlvB, found in Hafnia alvei strain MISC261 (19).

### Data availability.

This whole-genome shotgun project has been deposited in DDBJ/ENA/GenBank under the accession number JAEAGQ000000000.1. The raw sequences are available in the SRA under the accession number SRR14280049 and the BioProject accession number PRJNA682044.
